# Diabetic macular edema: it is more than just VEGF

**DOI:** 10.12688/f1000research.8265.1

**Published:** 2016-05-27

**Authors:** Michael A. Singer, Daniel S. Kermany, Jana Waters, Michael E. Jansen, Lyndon Tyler

**Affiliations:** 1 Medical Center Ophthalmology Associates, San Antonio, TX, USA; 2 University of Texas at Austin, Austin, TX, USA; 3 University of Texas Health Science Center, Austin, TX, USA

**Keywords:** macular edema, VEGF, Diabetic macular edema, Laser photocoagulation

## Abstract

Diabetic macular edema is a serious visual complication of diabetic retinopathy. This article reviews the history of previous and current therapies, including laser therapy, anti-vascular endothelial growth factor agents, and corticosteroids, that have been used to treat this condition. In addition, it proposes new ways to use them in combination in order to decrease treatment burden and potentially address other causes besides vascular endothelial growth factor for diabetic macular edema.

## Introduction

Diabetic macular edema (DME) is a common complication of diabetes and the leading cause of vision loss in adults ^1^. Macular edema occurs when high blood glucose damages the small capillaries supplying blood to the retina. This breakdown of blood vessels leads to the extravasation of blood and its solutes from the capillaries to the extracellular space under the macula, the central area of the retina, and causes it to thicken and swell (edema). The macula holds tightly packed cones that provide sharp central vision, enabling the person to see vivid detail and color. In this review, we briefly discuss the treatment options currently available for the treatment of macular edema and we review the rationale for emerging agents, many of which are currently being evaluated in clinical trials.

## Treatments

### Laser photocoagulation

Laser photocoagulation has been the gold standard for the treatment of DME for the past two decades. Laser treatment involves placing tiny laser burns within thickened areas of the retina in both direct (focal) treatment of microaneurysms and scattered spots in other areas of edema (grid). Laser photocoagulation involves the application of a precise and directed high-energy laser to the retina, and the heat generated as it is absorbed into the tissue causes clotting of the blood vessels and leads to the localized destruction of the tissue. These burns in the light-sensitive membrane in the back of the eye serve to destroy the diseased areas of the tissue and to seal off the damaging blood vessels that threaten vision.

This treatment for macular edema is very prevalent since it is a quick and cost-effective procedure that is usually completed after one session without the risk of endophthalmitis that an intravitreal injection poses. However, laser photocoagulation focuses on the symptoms of edema instead of addressing the retinopathy. The benefits to visual acuity are unremarkable, as there is only a 50% reduction in vision loss and vision already lost cannot be regained using laser photocoagulation. Lesions on the retina left by the destructive laser have been observed to expand over time. At 2 years, laser sears increased 50% per year and 4.6% a year afterwards, and 11 out of 203 patients experienced foveal encroachment. Still, laser photocoagulation is a highly effective treatment for macular edema and is still a feasible option for those unresponsive to anti-vascular endothelial growth factor (anti-VEGF) treatments ^2^.

### Anti-VEGF

The VEGF family is the most critical with regard to the pathogenesis of diabetic retinopathy owing to its signaling the induction of angiogenesis as well as increasing vascular permeability. Because of VEGF’s central role in the pathogenesis of DME, VEGF antagonists are a logical choice for therapy. The first anti-VEGF agent used for ophthalmology was pegaptanib (OSI Pharmaceuticals, Long Island, NY, USA) but was replaced by the development of ranibizumab (Genentech, Inc., South San Francisco, CA, USA). In the randomized clinical trial Protocol I, researchers observed that ranibizumab triples the mean change in visual acuity compared to the corticosteroid triamcinolone, and in 1 year the same steroid showed a visual acuity loss three times higher than that produced by the VEGF antagonist ^3^. In addition, the RISE and RIDE studies demonstrated that 39.2% of patients had 15-letter gains in visual acuity and a mean improvement of 12.4 letters vs. sham over 24 monthly injections and were the pivotal studies that allowed approval of ranibizumab by the US Food and Drug Administration ^4^.

Bevacizumab (Genentech, Inc., South San Francisco, CA, USA) is another VEGF antagonist that has come into widespread clinical use in the treatment of retinal disease. The BOLT study compared bevacizumab vs. macular laser in patients with DME. The bevacizumab arm gained a median of 9 ETDRS letters vs. 2.5 letters of laser treatment (P=0.005), with a mean gain of 8.6 letters for bevacizumab vs. a mean loss of 0.5 letters for laser. Forty-nine percent of patients gained 10 or more letters (P=0.001) and 32% gained at least 15 letters (P=0.004) for bevacizumab vs. 7% and 4% for MLT. The percentage who lost fewer than 15 letters in the laser arm was 86% vs. 100% for bevacizumab (P=0.03) ^5^.

Aflibercept is the most recent anti-VEGF medication approved to treat DME. In the randomized clinical studies VIVID and VISTA, researchers compared intravitreal aflibercept injections, which have recently demonstrated clinically equivalent efficacy to monthly ranibizumab in neovascular age-related macular degeneration, to laser monotherapy for the treatment of DME. The results of the trials demonstrated that aflibercept, given either every 4 weeks or every 8 weeks (after five initial monthly doses), is superior to laser and results in 10.7–12.4 letters gained at 1 year. In addition, 32–41% of patients gained 15 letters at 1 year as well ^6^. These visual acuity results indicate that a large portion of patients with DME may be effectively treated with dosing every 8 weeks compared to the monthly injections of other anti-VEGF agents.

However, it is widely accepted that the systemic use of anti-VEGF agents results in an increased risk for arterial thromboembolic events, obstructions of a blood vessel caused by a blood clot that has become dislodged from another point in the circulation, which can show a higher incidence of stroke among patients receiving this therapy. However, it is noteworthy that no significant increased rate of death, stroke, or myocardial infarction was seen in the RISE and RIDE or VIVID and VISTA groups.

### Predicting anti-VEGF efficacy

About 50% of patients with DME experience only a moderate reduction of edema and improvement in vision from VEGF antagonists alone. However, for most clinicians, it takes many months or years to determine the need for a switch in therapy. The EARLY analysis was a post hoc analysis of Protocol I of the diabetic retinopathy clinical research group ^7, 8^. This analysis looked at the two arms of patients who received ranibizumab and looked at vision at 12 months and 3 years. The study showed that patients could be divided into three groups: those who were good responders with 10 or more letters of improvement, those who were fair responders with 5 to 9 letters of improvement, and those who were suboptimal responders with 5 or fewer letters of improvement. The study showed that the best-corrected visual acuity (BCVA) response after three anti-VEGF injections (12 weeks) is a strong predictor of long-term BCVA response at 12 months and 3 years. This study demonstrated that physicians can recognize suboptimal DME responders much earlier in the treatment cycle and should consider different therapies in patients who are suboptimal responders.

### Corticosteroids

Mounting evidence exists to show that inflammation is a significant aspect of the pathogenesis of DME. Leukocytes in the blood release a variety of cytokines and chemokines that significantly increase vascular permeability, leading to more fluid build-up under the retina. These cytokines also carry VEGFs, which can aggravate and worsen macular edema by promoting angiogenesis ^9^.

Corticosteroids have shown the ability to lower inflammatory mediators and VEGF, while anti-VEGF therapy treats only the VEGF portion. Anti-VEGF treatment does not work for all patients; 50% of patients respond significantly and quickly, 25% of patients have an intermediate response, and 25% of patients do not respond to anti-VEGF treatment ^10^. Steroids have been shown to lower the central subfield thickness (CST) and improve visual acuity for suboptimal responders to anti-VEGF and pseudophakics. Corticosteroids also appear to be effective for both chronic and treatment-naive macular edema, while anti-VEGF therapy is seen to be a less effective treatment for chronic DME.

There are currently two approved corticosteroid therapies for DME: dexamethasone (Ozurdex, Allergan) and fluocinolone (Iluvein, Alimera). The MEAD study showed that dexamethasone intravitreal implant was able to improve vision in patients over a 3-year period. Patient vision improved overall by 4 letters and pseudophakic patients improved by 6 letters over a year period. In the MEAD study, there was a nearly 60% frequency of cataract surgery, and 40% of patients on steroids were later prescribed medication for intraocular pressure (IOP). However, patients’ increase in IOP usually peaked at 6–8 weeks and then returned to baseline by the end of 4 months, and one patient required IOP-lowering surgery ^11^.

The advantage of corticosteroid delayed delivery systems, such as dexamethasone intravitreal implants, is that patients require fewer treatments compared to the monthly injections required by most anti-VEGF agents. The newest steroid to be approved is the fluocinolone implant. The FAME study showed that the fluocinolone implant caused an improvement of ≥15 letters in 28.7% of patients in the study group vs. 18.9% in sham eyes and a 6-letter improvement in vision at 24 months. IOP medications were required in 42% of patients with seven patients requiring IOP-lowering surgery ^12^.

### Combination therapy

Although anti-VEGF medication remains the mainstay of therapy for DME, there are many cases for which anti-VEGF therapy alone is not adequate enough to control the macular edema. This should not be unexpected, as the clinical trials were able to significantly improve vision in fewer than 50% of patients ^4– 6, 13^. VEGF is a logical drug target that treats DME well, but it requires monthly retreatment to maintain efficacy and it does not address additional inflammatory cytokines upregulated in DME. It is in these patients that supplementing with therapies that work using a different mode of action may be of value. Diabetic maculopathy is a combination of both VEGF-mediated factors as well as inflammatory mediators. Corticosteroids decrease inflammatory cytokines and have a modest anti-VEGF effect, while anti-VEGF agents have a modest anti-inflammatory effect ^9^. Using a corticosteroid in combination with an anti-VEGF agent allows the patient to benefit with increased efficacy as well as increased duration of effect. As the category of sustained release steroids increases, the physician’s arsenal of managing these “hard to treat” patients increases as well.

A 12-month randomized study of eyes with persistent DME assessed the efficacy of a corticosteroid (dexamethasone) delivery system as an adjunct to the VEGF antagonist bevacizumab compared with continued bevacizumab monotherapy ^14^. After 12 months, it was observed that while ultimately there was no difference in vision, there were differences in vision at different monthly time points and the optical coherence tomography (OCT) CST was significantly better in the combination group, with a “sawtooth” effect ^14^. Subgroup analysis suggested that the greatest benefit of dexamethasone implant was in the group with the most bevacizumab injections prior to enrollment in the study ^14^. In conclusion, although visual acuity changes are not superior to continued bevacizumab monotherapy, dexamethasone combined with bevacizumab significantly improves visual acuity and significantly improves macular morphology in eyes with refractive chronic DME.

In another study, researchers explored the effect and safety of fluocinolone acetonide in chronic DME patients considered insufficiently responsive to available therapies with or without intravitreal corticosteroid therapy ^15^. The study covered 12 patients who received a single injection of fluocinolone acetonide and were followed for 6 months. Out of the 11 patients who completed the study, nine maintained or improved their best-corrected visual acuity from baseline, and the 11 patients experienced an average decrease in CST of 300.6 microns from baseline. However, there are no good data on combination therapy with the implant, but it may be appropriate for patients with suboptimal edema reduction with anti-VEGF monotherapy ^15^.

Combination therapy is a rational approach to battling DME, as it targets VEGF-mediated angiogenesis while combating the multiple factors in the inflammatory cascade. Combination therapy may be an alternative for patients who are unresponsive or have a suboptimal response to VEGF antagonists by providing a sustained duration of action, which translates into increased efficacy and convenience. However, there are potential adverse effects, such as cataracts and elevated IOP, that need to be considered.

## Abbreviations

BCVA, best-corrected visual acuity; CST, central subfield thickness; DME, diabetic macular edema; IOP, intraocular pressure; OCT, optical coherence tomography; VEGF, vascular endothelial growth factor.
